# On the Development of Virtual Reality Scenarios for Computer-Assisted Biomedical Applications

**DOI:** 10.1155/2018/1930357

**Published:** 2018-08-30

**Authors:** Eder H. Govea-Valladares, Hugo I. Medellin-Castillo, Jorge Ballesteros, Miguel A. Rodriguez-Florido

**Affiliations:** ^1^Facultad de Ingeniería, Universidad Autónoma de San Luis Potosí, 78290, San Luis Potosí, Mexico; ^2^Instituto Tecnológico de Canarias, Playa Pozo Izquierdo s/n, 35119, Las Palmas, Islas Canarias, Spain

## Abstract

The modelling of virtual environments and scenarios is an important area of research for the development of new computer-assisted systems in the areas of engineering and medicine, particularly in the area of biomechanics and biomedical engineering. One of the main issues while designing a virtual environment is the level of realism, which depends on the computing capacity and the level of accuracy and usefulness of the generated data. Thus, the dilemma is between the aesthetic realism and the information utility. This paper proposes a methodology to develop low-cost and high-quality virtual environments and scenarios for computer-aided biomedical applications. The proposed methodology is based on the open-source software Blender and the Visualization Toolkit libraries (VTK). In order to demonstrate the usability of the proposed methodology, the design and development of a computer-assisted biomedical application is presented and analysed.

## 1. Introduction

In the early days, scientific research was based on observations of natural and physical phenomena. However, in the last years, the research scope has included the modelling and simulation of physical phenomena by means of computer technologies. The sight has been the most developed sense in computer simulations and virtual environments, leading to the origin of the scientific visualization concept [1]. By means of scientific visualization, it is possible to transform mathematical data into 2D or 3D images or vice versa [2], allowing the communication and understanding of large amount of information efficiently. It also allows the visualization of physical phenomena that are not possible to visualize by common methods, such as experimental observations. Moreover, the representation of reality is also possible by means of animations, modelling, and graphic renderings.

Virtual reality (VR) can be described as a set of technologies that enable people to interact with a virtual environment beyond reality [3]. VR takes advantage of the computer technological development and scientific visualization to create a virtual world [4]. The use of VR has become very popular because it offers a high level of realism and immersion but requires advanced computing technologies capable of processing large amounts of scientific data and graphics [5]. VR has been used in different areas such as engineering, medicine, education, entertainment, astronomy, archaeology, and arts. In the area of medicine, virtual environments are created to enable the interaction with the human body anatomy [6]. The practice of medicine is a complex decision-making process that requires knowledge, experience, and manual abilities [7]; practitioner abilities are gained by training and experience, which is a slow process that may take several years. In order to get experience and abilities, a medical apprentice must be the protagonist of his/her training but considering as the main priority the avoidance of risks and unnecessary inconveniences for the patient [8]. Consequently, the use of VR and computer technologies in medicine has become an important tool for students and practitioners to understand and confirm concepts and to improve surgical skills and for experienced surgeons to make more precise diagnosis and plan the surgery [9–11]. One of the main applications of VR and computer technologies in medicine has been the development of computer-assisted surgery and simulation systems [12, 13].

In the area of engineering, VR applications include the design and evaluation of components and prototypes before construction [14–16], the manufacturing planning of components [12, 17–19], and assembly training [20]. On the other hand, VR applications in the area of art include virtual sculpting [21], reconstruction and preservation of buildings [22, 23], and development of environments, structures, and scenarios for the film industry [24].

A main issue of VR and computer-assisted applications is the design and development of the virtual environment (VE), which comprises the modelling of virtual objects, including their geometry and surface characteristics such as colours and textures. However, the amount of data to be processed by the VR application increases as the level of realism of the virtual environment increases, affecting the performance of the application. Thus, the dilemma is between the largest amount of data to increase the quality of the results, and the minimum use of computer resources with an acceptable level of realism.

This work presents a methodology to design and develop high-quality virtual scenarios with a high degree of realism for medical applications. The methodology is based on the open-source software Blender and VTK, leading to a low-cost development. The proposed approach is intended to be used in the development of computer-assisted biomedical applications, such as surgery planning, simulation, and training.

## 2. Literature Review

The solid modelling developed in the mid-1970s [25] is a set of mathematical principles for modelling objects, such as solid or hollow shapes delimited by a mesh, using computational methods [26], that is, creating digital models of physical objects of the real world. The main feature of solid modelling is that it is focused on the surface characteristics of the object. The constructive solid geometry (CSG) considers the modelling of solid objects using Boolean operations, which is useful for tasks requiring mathematical precision [27, 28]. On the other hand, Boundary Representation (B-Rep) [29] connects vertices with lines to create faces, allowing the generation of complex geometries where the level of accuracy depends on the amount of elements in the mesh [27, 28].

Several applications of solid modelling in the area of medicine can be found in the literature, for instance, the 3D modelling of a femur by using the software called 3ds Max [30]. One example of designing virtual scenarios for surgical planning was presented by Domínguez-Quintana et al. [31]. Snyder et al. [32] presented an investigation to compare the impact of training with or without supervision using a virtual reality surgical simulator of laparoscopy and endoscopy. Debes et al. [33] compared the training effectiveness between a virtual simulator and a training video in laparoscopic surgery. A successful virtual environment requires that virtual parts behave as the parts in the real world [34].

Virtual reality (VR) technologies can be used to enhance the performance of surgical simulators by providing a virtual environment where users can get the feeling of immersion in a real environment, in addition to more intuitive cues such as collisions between virtual objects, collisions with obstacles, friction, inertia, restitution, 3D rendering, and sound [35]. Moreover, virtual environments can be improved by incorporating haptic technologies to provide the user with the sense of touch. Haptics allows natural manipulation of virtual objects by enabling the user with the feeling of collisions, forces, weight, and inertia of virtual objects. In this way, haptic-enabled computer-assisted medical applications are more intuitive, accurate, and efficient than conventional computer-aided systems in medicine [11].

In general, a VR application in medicine comprises five main modules: (1) model reconstruction module, to generate 3D models from medical data such as CT and MRI images, (2) visualization module, responsible for the graphics rendering of the virtual environment, (3) manipulation module, to provide the interaction between the user and the virtual environment, (4) simulation module, responsible for the physical based behaviour of the virtual environment and objects, and (5) data module, responsible for processing, analysing, and exporting the medical data.

There have been several research works reported in the literature focusing on the development and analysis of computer-aided VR applications in medicine. However, few works have addressed the development of virtual environments for such applications [10]. A virtual environment has a great impact on the performance of the application; therefore, its importance is high. Sometimes, it is necessary to sacrifice the level of realism to prioritize the data processing and time response of the system. Nowadays, there are several tools such as modelling software, computers with high computing power, and measurement devices, to develop virtual scenarios. However, these tools can be very expensive and limited to those users with financial capability to such tools. In addition, very few works in the literature have focused on reducing the costs of developing virtual environments.

## 3. Methodology

**Figure 1:**
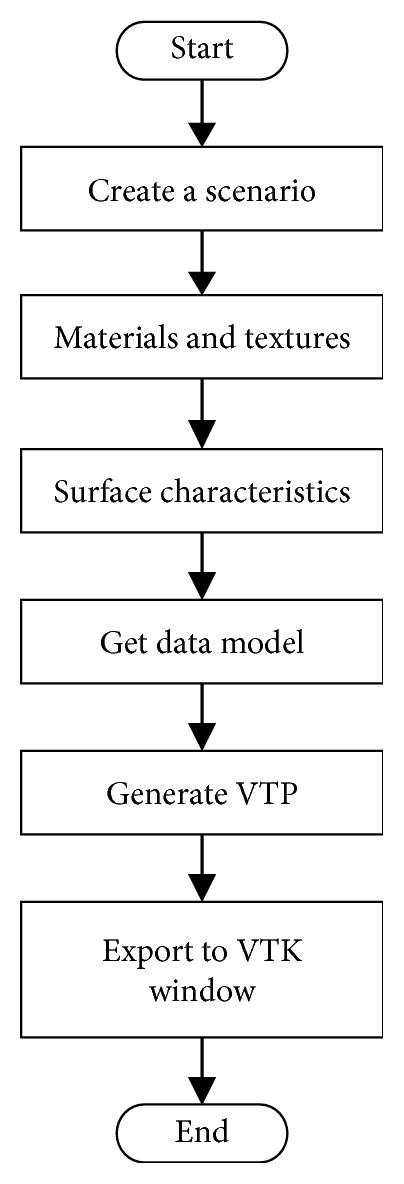
Methodology to create virtual reality scenarios.

A new methodology to create virtual reality scenarios (environments) is proposed as shown in Figure 1. This methodology has been implemented using the open-source software Blender 2.49, the Visualization Toolkit VTK 5.6, and the Python 2.7 programming language in Windows operating system.

**Figure 2:**
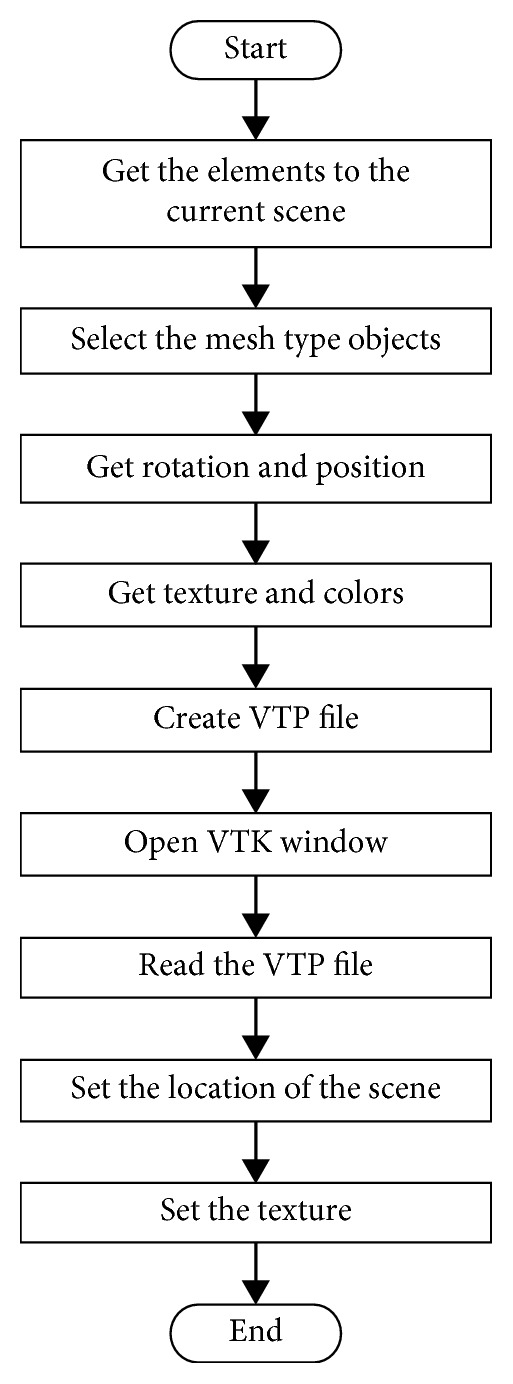
Algorithm to export the Blender scene to a VTK application.

The main steps of the proposed methodology are as follows:

1. *Create scene*. Modelling starts in Blender and the first step is to generate the virtual models to be placed on the stage. 3D models can be imported or created using the commands, primitive objects, and Boolean operations of Blender.
2. *Materials and textures*. Textures are then assigned to each element of the models. The texture corresponds to the type of material and the visual aspect that they have in the real world. This process of adding textures is done by using standard images or by assigning colour to each part in Blender.
3. *Surface characteristics*. To boost realism, it is necessary to provide the VE with visual features like lighting, shadows, reflections, transparencies, and more. This process can be carried out in Blender.
4. *Get data model*. Once the scene has been completed, the next step is to extract the information corresponding to orientation, rotation, and location of each object in the scene.
5. *Generate VTP*. In this step, the virtual scenario is converted into a VTP file. The VTP (VTK Polygonal Data) is a VTK file that contains the polygonal data of the 3D model.
6. *Export to VTK window*. Finally, the VTP file is used to import all the elements of the scene into a VTK window in the external application being developed. The algorithm to export the Blender scene to a VTK window of the external application is shown in Figure 2.

In order to show the level of realism that can be obtained using the proposed methodology, two virtual scenarios were developed. These scenarios are described in the next paragraphs.

### 3.1. Jaw Articulator

**Figure 3:**
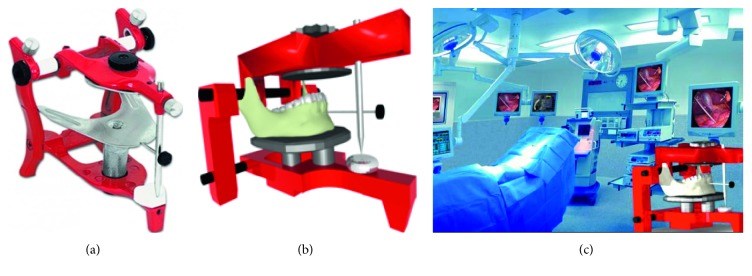
Jaw articulator. (a) Real articulator. (b) Virtual articulator. (c) Virtual scenario.

The first medical virtual scenario corresponds to a jaw articulator, which is a mechanical device that represents the human jaw joints, and that is used to simulate and adjust the motion of the jaw physical models. The jaw articulator is used for oral and maxillofacial surgery planning.  Figure 3(a) shows the real-world articulator used as reference. Each part of the articulator was created using primitive objects and Boolean operations in Blender 2.49. Texture characteristics such as colour and visualization properties such as transparency level, reflection, shadows, and so on were also added to the different parts of the virtual model, Figure 3(b). A 3D model of a human jaw was obtained from medical image reconstruction and imported into the virtual scenario where the articulator was created. Also an image type texture was added to simulate the real bone texture. Finally, a smoothing filter was applied to obtain a smooth surface free of imperfections due to the mesh.  Figure 3(c) shows the virtual jaw articulator and the final virtual scenario corresponding to a hospital.

From Figure 3, it can be observed the lighting effects on the elements' surface, the texture applied to the mandible, and some surface properties to simulate the light reflection. It can also be observed that there are some elements that simulate different types of materials, such as plastics and metals, resulting in objects with transparency and very high gloss. Moreover, metallic elements give a more realistic visualization.

### 3.2. Surgical Simulator

**Figure 4:**
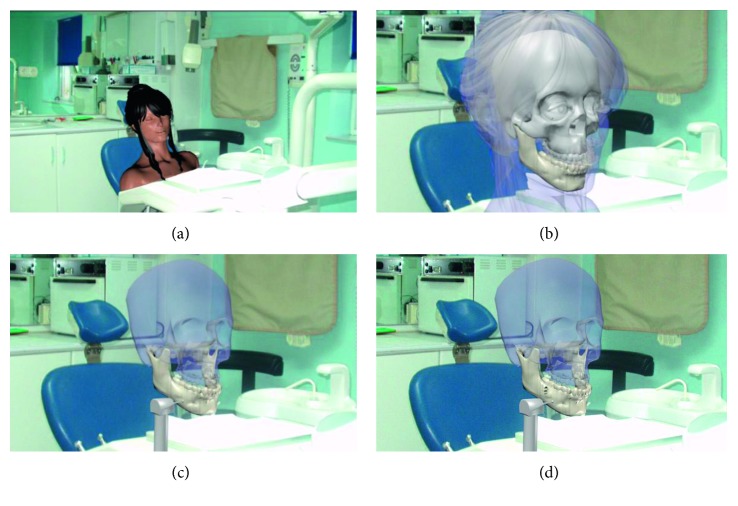
Medical surgical simulator. (a) Dental room. (b) Transparent layers of virtual patient. (c) Virtual tool for bone cutting. (d) Results of virtual bone cutting.

Biomedical engineering makes use of virtual environments to develop surgical simulators for planning and training various clinical procedures. Thus, the second virtual scenario corresponds to an orthognathic surgery simulator to perform dental surgeries or procedures in a virtual dental office environment.  Figure 4(a) shows the virtual scene corresponding to a dental room with a patient sitting in a dental unit. Textures, lights, and images can be used to increase the level of realism. Transparency can be also used to observe internal details such as the bone structure, Figure 4(b). Dental surgical tools, such as mills or drills, can be modelled and added to the virtual scenario to perform virtual cuts on the patient jaw bone, Figure 4(c). The advantage of this type of virtual scenarios is that the practitioner can perform the surgical procedures as many times as necessary in order to practice or to plan the real surgery procedure.  Figure 4(d) shows the footprint of the virtual cut performed on the virtual mandible.

## 4. Case Study

In order to show the details and usability of the proposed methodology for the development of VR scenarios for computer-aided biomedical applications, a Virtual Osteotomy Simulator System (VOSS) for 3D osteotomy simulation and training was developed and evaluated.

### 4.1. System Description

**Figure 5:**
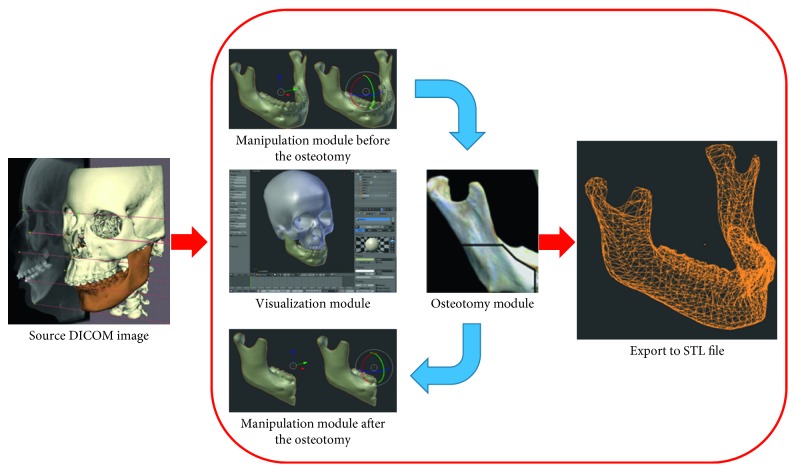
VOSS architecture.

The VOSS general architecture is shown in Figure 5 and comprises four main modules:

1. Visualization module, responsible for carrying out the graphic rendering of virtual objects, tools, and virtual environments
2. Osteotomy module, responsible for computing bone cuts and enabling virtual osteotomies
3. Manipulation module, to allow the 3D free-form movement and manipulation of virtual objects and surgical tools
4. Data exportation module, to export any information regarding the osteotomy simulation (e.g., STL models)

The VOSS was implemented using Python 2.7 and Blender 2.59 in a Workstation with a dual-core AMD processor (3.0 GHz/dual core), 4 GB of RAM, and a NVIDIA Quadro FX3500 PCI Express graphics card. The main capabilities of the VOSS are as follows:

- Virtual reality environment and real-time response
- 3D visualization of anatomical models and tools, including textures and transparency
- 3D free manipulation and interaction of virtual cutting tools, bones, and bone fragments
- Simulation of single and multiple osteotomies
- Free-form cutting path to perform osteotomies
- Free camera manipulation
- Automatic scaling of models.

**Figure 6:**
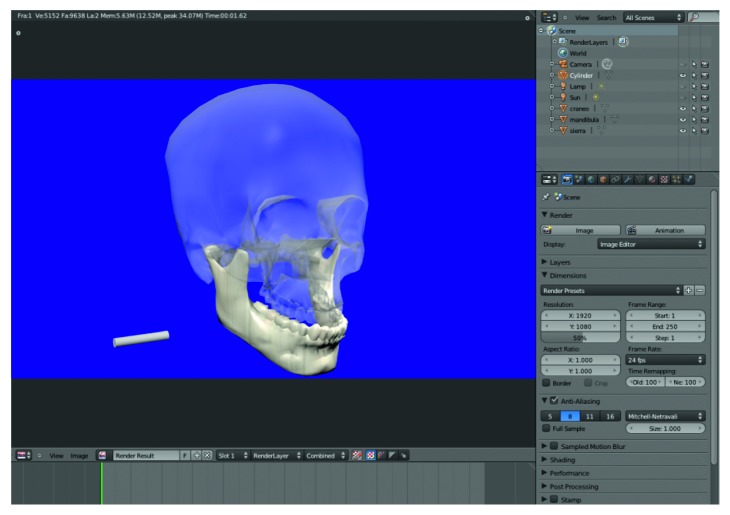
VOSS graphic user interface (GUI).

The GUI of the VOSS is shown in Figure 6. The biomodels can be imported into VOSS as STL or 3DS file formats, which can be generated from medical images (e.g., DICOM images) or 3D scanning.

### 4.2. Virtual Scenario

**Figure 7:**
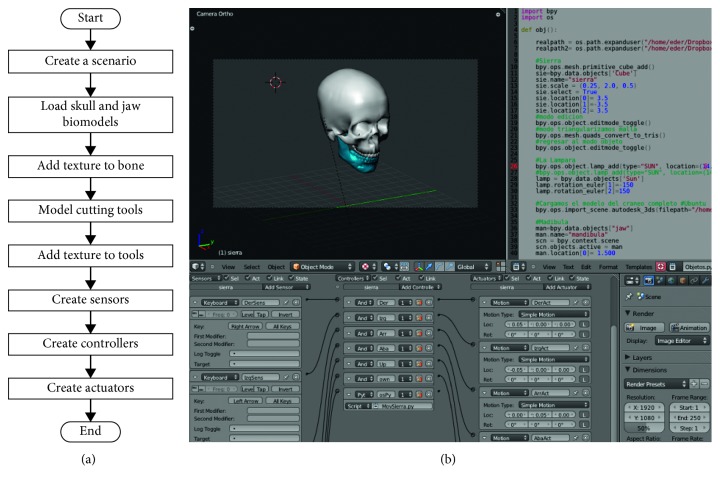
VOSS virtual reality environment. (a) Methodology. (b) Implementation.

Figure 7 shows the particular methodology used to define the virtual reality environment of the VOSS. This methodology comprises the following steps in Blender:

1. Create a scenario. Generate the virtual environment (lights, cameras, and background image)
2. Load skull and jaw biomodels. Import the skull and jaw models as STL or 3DS file formats
3. Add texture to bone. Add texture to the skull and jaw bones by means of images. Since the skull is used only as reference and visual support, its texture can be set as transparent
4. Model cutting tools. Create the surgical cutting tools (saw and drill) in Blender or import them as STL or 3DS file formats
5. Add texture to tools. In order to reproduce the real appearance of surgical cutting tools, provide them with texture
6. Create sensors. Generate sensors for the user to control and manipulate objects in the virtual environment by means of the keyboard and/or mouse buttons
7. Create controllers. Generate controllers to specify the action to be executed after the activation of a sensor
8. Create actuators. Generate actuators to perform the movement or manipulation of the virtual objects according to the sensors. Movements can be either linear or rotational.

The addition of textures to biomodels or tools can be made by means of images with standard file formats (e.g., bmp, jpg, jpeg, and png). The movement and manipulation of virtual models in VOSS is made by means of a numeric keypad, an alphanumeric keypad, a standard computer mouse, or a 3D computer mouse. These movements are defined by sensors, controllers, and actuators, which are created in the Logic mode of the Game Engine in Blender. A sensor is a function for the user to control objects in the virtual environment by means of the keyboard and/or mouse buttons. On the other hand, a controller is a function used to define the action to be executed after the activation of a sensor. Finally, an actuator executes the movements of the virtual objects according to the sensors.

### 4.3. Virtual Osteotomy

**Figure 8:**
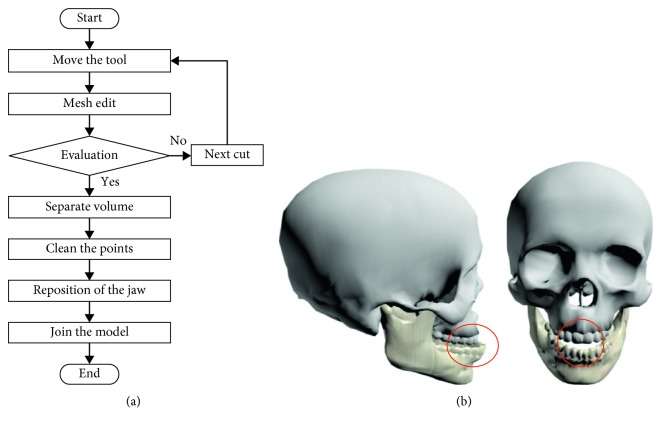
Virtual osteotomy. (a) Procedure. (b) Virtual model with malformation.

The virtual osteotomy procedure implemented in the VOSS corresponds to a Bilateral Sagittal Split Osteotomy Ramus Mandibular (BSSROM) of a human mandible. The aim is to perform bone cutting operations on virtual models of human jaws, that is, to simulate the work of a maxillofacial surgeon when correcting bone malformations.  Figure 8 shows the general virtual osteotomy procedure in VOSS.

**Figure 9:**
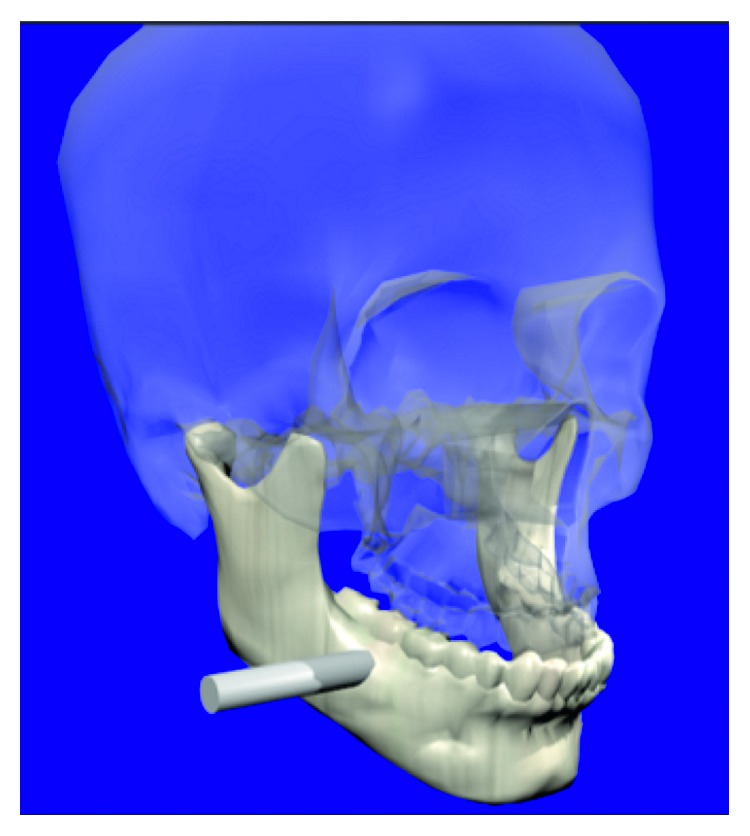
Initial positioning of the tool.

**Figure 10:**
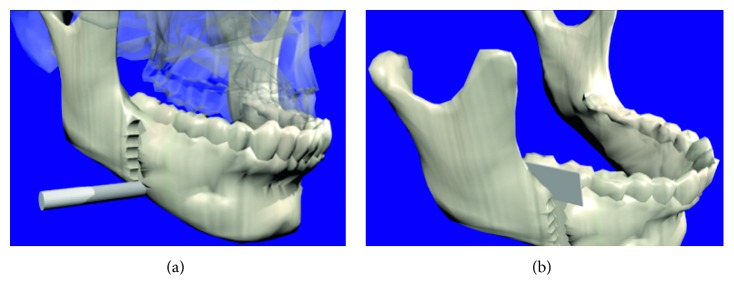
Cutting process. (a) Drilling. (b) Sawing.

**Figure 11:**
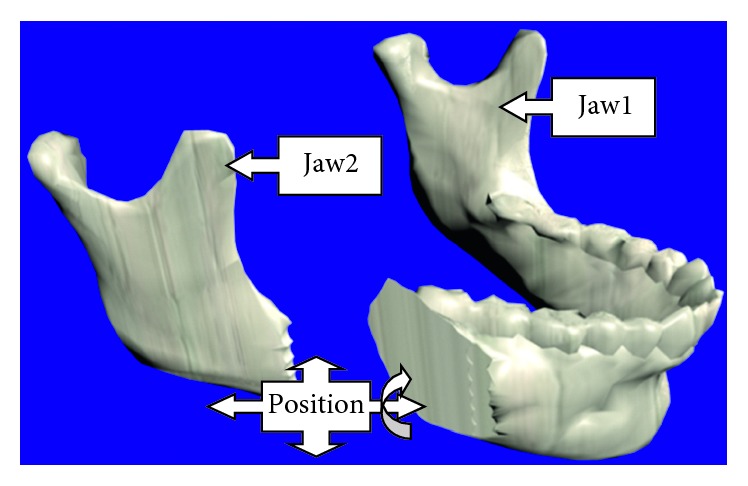
Repositioning of mandible fragments, jaw 1 and jaw 2.

The osteotomy procedure begins by selecting a cutting tool and placing it at the position where the first cut is meant to be made, Figure 9. Once the tool is positioned, the cut is performed and it can be repeated as many times as necessary to make a longitudinal cut along the jaw. The user is able to freely move the tool in any 3D path while performing the cut.  Figure 10(a) shows the simulation of a vertical cut using a drill, while Figure 10(b) shows the simulation of a cut operation using a sagittal saw. Similar to the real procedure, the virtual jaw can be separated into two fragments (jaw 1 and jaw 2), which can be moved or manipulated independently.  Figure 11 shows the bone fragments after the bone separation. The virtual cutting and separation of biomodels are performed by means of the Boolean operations in Blender.

**Figure 12:**
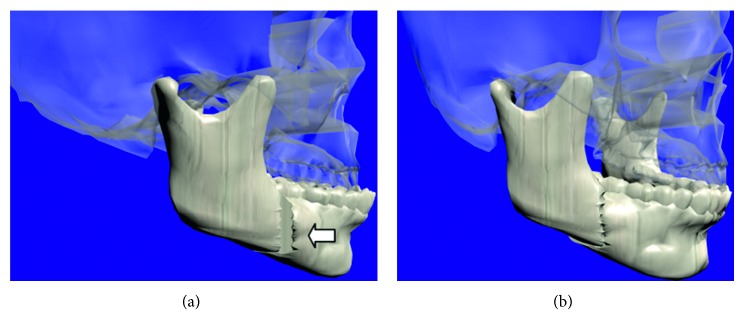
Final step of the virtual osteotomy. (a) Relocation of mandible fragments. (b) Joining of the mandible.

After the mandible splitting, the user is able to manipulate and relocate the jaw fragments in order to reduce or eliminate the bone defect or malformation. Once the mandible fragments are relocated at the correct position, a Boolean operation is carried out to join the jaw fragments.  Figure 12 shows the last movement and final relocation of the mandible.

### 4.4. Virtual Osteotomy Training

A biomedical application to evaluate the proposed virtual osteotomy approach as a training tool was developed in the programming language C++ using the Microsoft Foundation Classes (MFC) of Visual Studio 2010, the Visualization Toolkit libraries (VTK) for graphics rendering, and the H3DAPI haptic rendering software development platform for the manipulation of virtual objects. The application provides the user with force feedback by means of a haptic device. Additionally, the application supports different types of haptic devices, including the Phantom Omni from Sensable and the Falcon from Novint.

**Figure 13:**
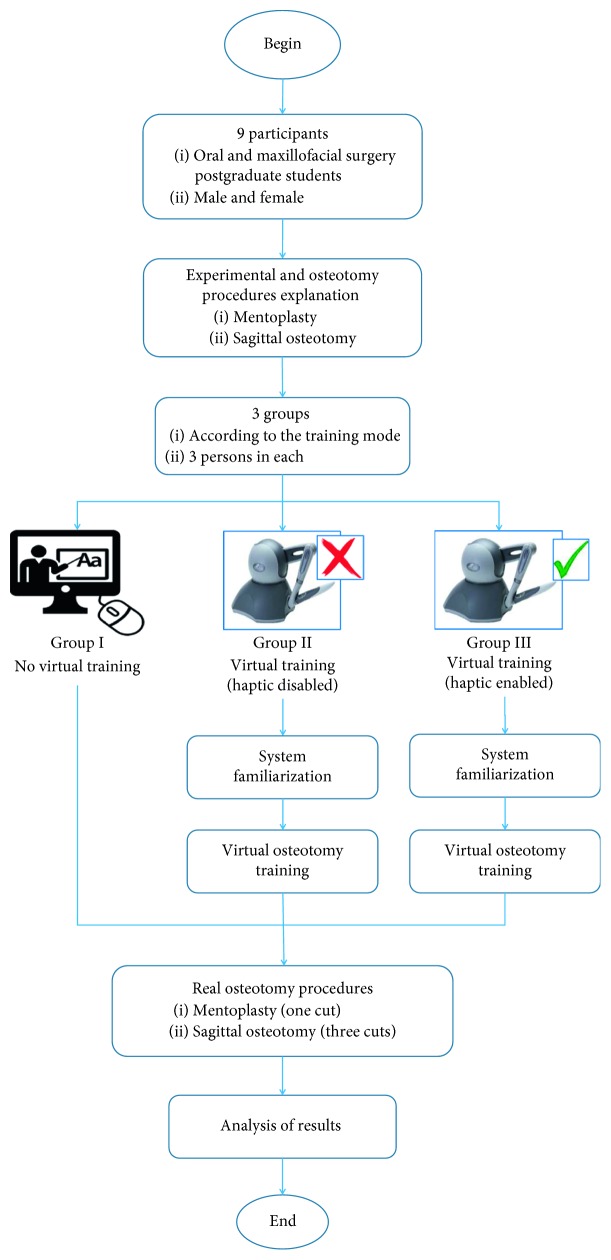
Experimental methodology to evaluate the virtual osteotomy training.

The overall experimental methodology used to evaluate the virtual osteotomy training is shown in Figure 13. A total of nine students of the Oral and Maxillofacial Surgery Postgraduate Program of the “Hospital Central Dr. Ignacio Morones Prieto” in San Luis Potosi, Mexico, were selected. These participants were selected because previous knowledge of the maxillofacial surgical procedures was required. The nine participants were divided into three groups with three persons in each group:

- *Group I*. No virtual training. This group of participants carried out the real osteotomy procedure without previous virtual training.
- *Group II*. Virtual training without force feedback. Before carrying out the real osteotomy procedure, this group of participants undertook virtual training but with no force feedback.
- *Group III*. Virtual training with force feedback. Before carrying out the real osteotomy procedure, this group of participants undertook virtual training with force feedback.

**Figure 14:**
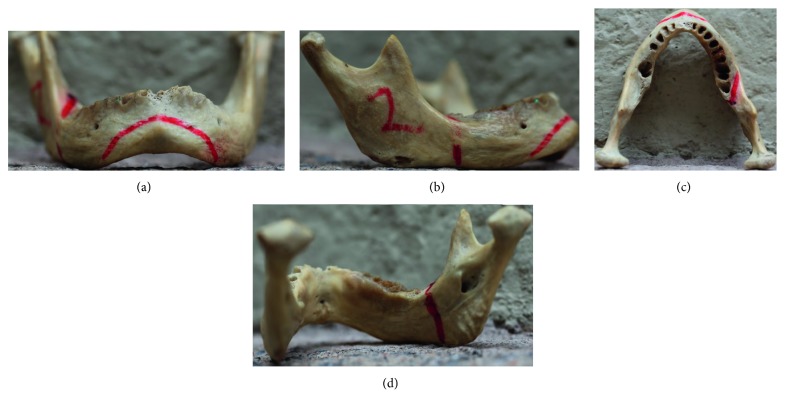
Osteotomy procedures marked by an expert on a real human mandible. (a) Mentoplasty. (b) Sagittal exterior. (c) Sagittal superior. (d) Sagittal interior.

Two different osteotomy procedures were considered in the evaluation: mentoplasty (or chin cut) and sagittal osteotomy (or branch cut) of a human mandible. The mentoplasty procedure comprises one main cut on the chin, while the sagittal osteotomy procedure comprises three cuts: sagittal exterior, sagittal superior, and sagittal interior.  Figure 14 shows these four cuts marked by an experienced maxillofacial surgeon on a real human mandible. These cutting trajectories were used as reference for evaluating the usability of virtual training.

At the beginning, all participants were informed about the general background related to the experiments, the conditions in which they would be working, and the experimental procedure. Then, all participants received a verbal explanation about the osteotomy procedures under consideration and the cuts required, allowing them to ask questions and receive further explanation. Participants of Groups II and III were instructed on the use of the virtual system and the virtual osteotomy procedure, given them the opportunity to familiarise themselves with the system for twenty minutes before undertaking virtual training.

Virtual osteotomy training for participants of Groups II and III consisted in the realization of the two osteotomy procedures in the virtual environment. Participants of Group II were able to manipulate the virtual surgical tools by means of the haptic device but without receiving force feedback, whereas participants of Group III did receive force feedback during the manipulation of the virtual surgical tools.

**Figure 15:**
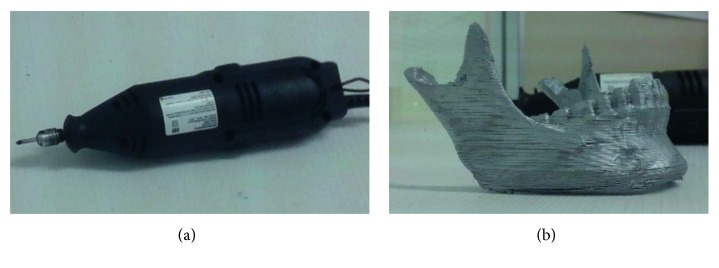
Real osteotomy implements. (a) Manual high-speed milling tool. (b) Physical jaw.

Virtual training was carried out on virtual models in the virtual environment by means of a Phantom Omni haptic device. On the other hand, the real osteotomy procedures were carried out on physical 3D prototypes, made of PLA in a 3DTouch printer from 3D systems®, and using a conventional high-speed milling tool, as shown in Figure 15.

### 4.5. Results and Discussion

**Figure 16:**
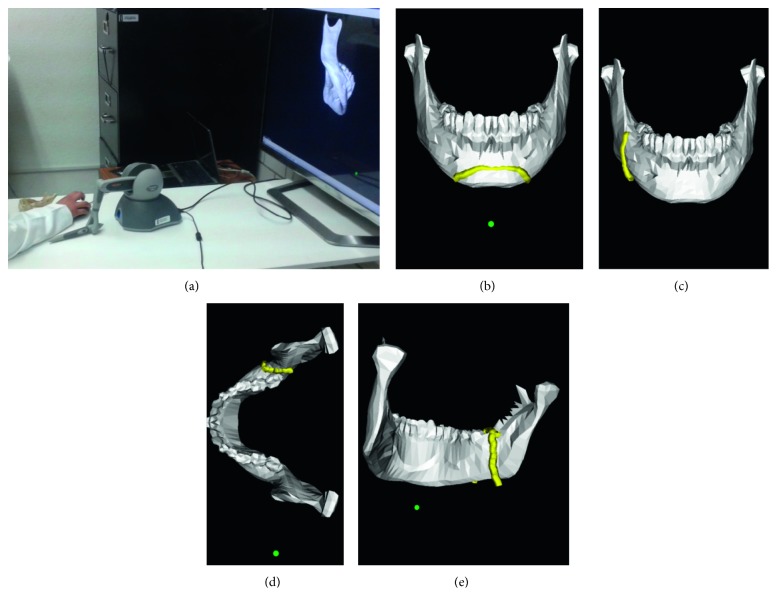
Virtual osteotomy training. (a) Participant. (b) Mentoplasty. (c) Sagittal exterior. (d) Sagittal superior. (e) Sagittal interior.

**Figure 17:**
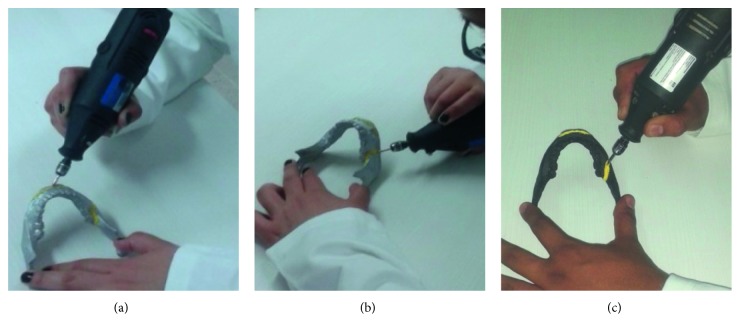
Real osteotomy procedure. (a) Mentoplasty. (b) Sagittal exterior. (c) Sagittal superior.

Figure 16 shows the results of the four virtual osteotomies performed by one participant of Group II. On the other hand, Figure 17 shows the real osteotomy procedure being performed by one participant of Group I.

**Figure 18:**
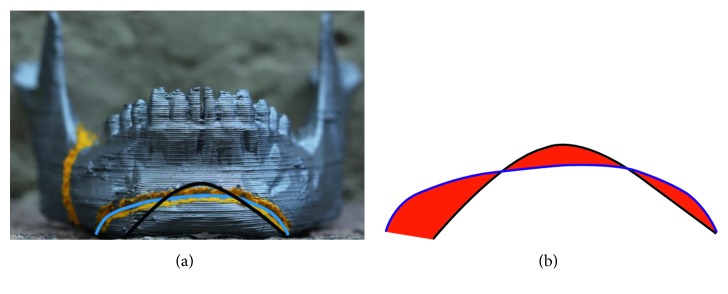
Error evaluation (black line: ideal trajectory, blue line: participant trajectory). (a) Front picture. (b) Error measurement (red area).

To evaluate the effectiveness of virtual training, all participants of Groups I, II, and III were observed during the real osteotomy procedure executions, and the time to complete the task was measured for all participants. The cutting error was also evaluated by comparing the cutting trajectory performed by each user, with the “ideal” cutting trajectory performed by an experienced maxillofacial surgeon, as shown in Figure 18(a). The cutting error was defined as the difference between the two trajectories, red area of Figure 18(b). This error was quantified in a CAD software.

Table 1 summarizes the results obtained for Groups I, II, and III during the real osteotomy procedures. The values reported in this table correspond to the average values obtained by each group of participants. From these results, it is observed that participants of Group I showed average task completion times of 432 s and 389 s for the mentoplasty and sagittal real osteotomy procedures, respectively. On the other hand, participants of Group II performed these procedures in 308 s and 317 s, respectively, and participants of Group III carried out these real tasks in 120 s and 241 s, respectively. These results suggest that participants that undertook virtual training first (Groups II and III) had superior performance, in terms of time, than those who did not train (Group I). In other words, participants who trained virtually completed the real osteotomy procedures faster than those who did not train. Moreover, participants who virtually trained with haptic force feedback (Group III) completed the real osteotomy procedures faster than those who virtually trained but without force feedback.

Regarding the cutting error, participants of Group I exhibited an average cutting error of 24.2% and 27.7% for the mentoplasty and sagittal real osteotomy procedures, respectively, whereas participants of Group II exhibited an average cutting error of 14.6% and 21.2%, respectively, and participants of Group III obtained an average cutting error of 6.4% and 4.9%, respectively. These results clearly evidence than participants of Group II and Group III, who undertook a virtual training period first, achieved a more accurate mandible cutting during the real osteotomy procedure than those who did not train first (Group I). Furthermore, participants of Group III who trained with force feedback exhibited smaller cutting errors than participant of Group II who trained but without force feedback.

Therefore, it can be said that the proposed virtual osteotomy training procedure is a feasible approach to improve the performance and skills of the participants. A faster and more accurate osteotomy procedure was achieved by subjects who undertook virtual training first than those who did not train virtually first. Additionally, the use of haptic force feedback during virtual training has showed to enhance the virtual training procedure; a better performance is achieved by subjects that train with haptic force feedback than subjects that train with no haptic force feedback. Thus, the usability of the proposed methodology to design and develop virtual scenarios for biomedical applications has been demonstrated.

## 5. Conclusions

A new methodology to develop virtual reality environments for biomedical applications has been presented in this paper. This methodology represents a low-cost solution for the development of virtual environments with a high level of realism and with physical characteristics very close to the real devices. A case study corresponding to a virtual osteotomy simulator was developed using the proposed methodology. The results of this virtual osteotomy training biomedical application have demonstrated the effectiveness of the virtual system; users increased their abilities and skills to perform real osteotomy procedures. Therefore, the usability of the proposed methodology and approach has been validated. Future work considers a more comprehensive analysis of the effect of biomedical virtual training on the performance of subjects. This analysis will include a larger number of participants, modelling and rendering of real cutting forces, and comparison with traditional training.

## Figures and Tables

**Table 1:** Time and error results of the real osteotomy procedures.

Participants	Mentoplasty		Sagittal	
	RO time (seconds)	Cutting error (%)	RO time (seconds)	Cutting error (%)
Group I	432	24.2	389	27.7
Group II	308	14.6	317	21.2
Group III	120	6.4	241	4.9

RO: Real Osteotomy.

## Data Availability

The datasets generated during and/or analysed during the current study are available from the corresponding author on request.
